# The Molecular Effects of SGLT2i Empagliflozin on the Autophagy Pathway in Diabetes Mellitus Type 2 and Its Complications

**DOI:** 10.1155/2022/8337823

**Published:** 2022-10-19

**Authors:** Ranin Saad, Hagar Tadmor, Offir Ertracht, Nakhoul Nakhoul, Farid Nakhoul, Farber Evgeny, Shaul Atar

**Affiliations:** ^1^Diabetes & Metabolism Lab, Baruch Padeh Poriya Medical Center, Israel; ^2^Cardiovascular Laboratory, Medical Research Institute, Galilee Medical Center, Nahariya, Israel; ^3^The Ophthalmology, Israel; ^4^Azrieli Faculty of Medicine, Bar Ilan University, Safed, Israel; ^5^The Cardiology Department, Galilee Medical Center, Nahariya, Israel

## Abstract

**Background:**

Type 2 diabetes mellitus (T2DM), especially hyperglycemia, is associated with increased glucose cell toxicity and oxidative stress that can lead to irreversible damage in the kidney such as diabetic nephropathy (DN). Autophagy plays a key role in the degradation of damaged intracellular proteins in order to maintain intracellular homeostasis and cell integrity. The disturbance of autophagy is involved in the pathogenesis of diabetic nephropathy. We aim to investigate the molecular effect of sodium-glucose transporter 2 inhibitor (SGLT2i) on the expression of ATG5 and its downstream collaborator LC3-II in diabetic nice model. *Material and Methods*. We used eight weeks old male mice: twenty C57BL/6 wild type (C57BL/6), twenty BTBR ob/ob (DM), and twenty BTBR ob/ob that were treated with empagliflozin (DM+EMPA), FDA approved SGLT2i. Lysate from murine renal cortex was analyzed by Western blot and immunohistochemistry. ATG5, LC3B, and fibronectin expression were analyzed in murine kidney tissues. All mice were sacrificed 13 weeks after the beginning of the experiment.

**Results:**

Histological and Western blot analyses reveal decrease ATG5, LC3-II, and fibronectin levels at renal specimens taken from DM mice. EMPA treatment reduced T2DM mice body weight and blood glucose and increased urine glucose. Further, it upregulated all of the abovementioned proteins.

**Conclusions:**

Hyperglycemia reduces LC3-II and ATG5 protein levels which contribute to deficiencies in the autophagy process, with development and progression of DN. SGLT2i significantly reduces progression of DN and onset of end-stage renal disease in T2DM patients, probably through its effect on autophagy.

## 1. Introduction

Diabetes mellitus (DM) type 2 (T2DM) is a metabolic disease characterized by chronic hyperglycemia, resulting from insulin deficiency, insulin resistance, or both [1]. Chronic hyperglycemia is associated with disruption and alterations in carbohydrate, lipid, and protein metabolism [2–4], with cellular damage in different organs such as the kidney, heart, and retina [5, 6]. Diabetic nephropathy (DN) is one of the common complications of diabetes. Above 50% of T2DM patients developed DN during their life. DN is characterized by renal functional and structural changes, such as glomerular mesangial expansion, accumulation of extracellular matrix (ECM), glomerular hypertrophy, and thickening of glomerular and tubular basement membranes with glomerular sclerosis, all lead to decrease in glomerular filtration rate [4]. The final stage of DN is end-stage renal disease and renal replacement therapy that lead to increased morbidity and mortality [3].

Numerous risk factors are associated with the development and progression of DN among them extended duration of DM, arterial hypertension, obesity, and hyperlipidemia, with most being modifiable by appropriate treatment. Other contributing factors, such as genetic factors including haptoglobin genotype, cannot be modified [5–9]. Nevertheless, hyperglycemia is of the greatest importance.

Previous studies demonstrated that hyperglycemia increases renal renin angiotensin aldosterone system (RAAS) activation, reactive oxygen species (ROS) generation, inflammation [10–13], and fibronectin expression [5–10]. All might activate several biochemical pathways, which can aggravate glumerulosclerosis, tubule-interstitial fibrosis, and tubular cells apoptosis, leading to DN disease. Nevertheless, different therapies including the common RAAS inhibitors are not protective enough to prevent DN progression [14–18].

Autophagy is a catabolic mechanism that involves lysosomal-dependent degradation of unnecessary or dysfunctional intracellular components. Autophagy preserves cells and tissues maintenance by replacing damaged cellular components and promotes cell's metabolic homeostasis. The process starts with the formation of a cellular component named autophagosome, by autophagy-related genes (Atg) proteins such as ATG5 and LC3, and finalizes by its fusion with lysosome and intraphagosome lysosomal degradation of cellular organelles to their basal components [13–19]. It was shown that autophagy has an essential role in maintaining kidney's cellular homeostasis, especially in podocytes and proximal tubular cells [20–24]. Under pathological condition, intensified activity of autophagy is required to maintaining podocytes and other renal cells [21, 22].

Studies suggest that both hyperinsulinemia and hyperglycemia inhibit autophagy activity in proximal tubular cells and podocytes, by inducing hyperactivation of mTORc1, an autophagy-regulated protein (20). Hyperglycemia suppresses the expression of LC3, Atg12-5, and Beclin-1, to inhibit the formation of the autophagosome membrane [25, 26]. Thus hyperglycemia alters autophagy activity and contributes to diabetes-related podocyte and tubular cell damage.

Empagliflozin (EMPA), FDA approved for cardiovascular protection [27, 28], is a renal sodium glucose cotransporter type 2 inhibitor (SGLT2i). It was seen that EMPA is highly effective in controlling blood glucose and HbA1c levels, increases glucose excretion, and slows the progression of renal injury [27–30]. Furthermore, it also improves blood pressure and decreases body weight (BW) and albumin secretion and slows the progression of renal failure [30–34]. Recent studies indicate that SGLT2is delay the progression of chronic kidney disease (CKD) in T2DM patients and cardiovascular morbidity and mortality by decreasing oxidative stress and apoptosis [32–38]. However, the precise mechanisms by which EMPA protect renal cells structure and function is not fully clear. In this study, we hypothesize that SGLT2i exerts its renal beneficial effects by affecting autophagy level. Thus, this study was designed to investigate the molecular effect of SGLT2i on the expression of ATG5/LC3-II autophagy key proteins in diabetic nice model.

## 2. Methods

### 2.1. Animal Model

Male BTBR ob/ob mice and C57BL/6 were purchased from Jackson Laboratory (Bar Harbor, ME, USA). Animals were group-housed and maintained on a 12 h/12 h light/dark cycle with *ad libitum* access to food and water. The animals were treated in accordance with NIH Animal Welfare Guideline (ed. 2011), and the Azrieli Faculty of Medicine, Bar-Ilan University, Institutional Animal Care and Use Committee (IACUC) approved all procedures [Ethical #48-07-2019]. BTBR mice with the ob/ob leptin-deficiency mutation were used as T2DM model, as they develop severe DM, which is presented in hyperglycemia, and DN [47-49] C57BL/6 mice were used as healthy control. Eight-week old mice were randomly divided in three groups: Group one C57/BL/6 as control (*n* = 15) and group two BTBR ob/ob vehicle as (DM), and third group BTBR ob/ob that were treated with EMPA (DM+EMPA).

Mice body weight was monitored once a week on a calibrated scale with a sensitivity of 0.01 mg. Water intake (ml/day), food consumption (gr/day), blood glucose (Glucometer, Atcu-Check, Roch), and daily urine production (ml/day) were measured. Urine samples were analyzed for glucose, protein, and creatinine excretion per day. All mice were sacrificed 13 weeks after the beginning of the experiment by ketamine-xylazine overdose injection. At sacrifice, kidneys were harvested, one kidney was flashed frozen in liquid nitrogen and transferred to -80°C until analysis, and the second kidney was submerged in 4% paraformaldehyde for histological analysis.

### 2.2. EMPA Administration

EMPA was purchased from DA-TA Biotech, USA. EMPA powder was diluted in water (1 mg/kg mouse) and administrated to the mice, as was published in another study, via drinking water for a period of 12 consecutive weeks, and drug concentration in drinking water was adjusted weekly to the BW [32–36].

### 2.3. Proteins Extraction and Western Blot Analyses

Mice kidneys were homogenized and incubated with radio-immunoprecipitation assay (RIPA) lysis buffer (Cell Signaling, USA) containing proteases and phosphatases inhibitors (Sigma, Israel) in ratio of 1 : 100 and 1 : 1000, respectively. Kidney's lysate was centrifuged, and the supernatant containing total proteins was subjected to sodium dodecyl sulfate-polyacrylamide gel electrophoresis (SDS-PAGE) and transferred to polyvinylidene difluoride membranes. Membranes were then blocked with 5% dry skim milk (Bio-Rad, CA, USA) in Tris-buffered saline with Tween 20 at room temperature and washed and incubated with primary antibodies (Anti-APG5L/ATG5 ab109490 [EPR4797] 1 : 1000; Anti-LC3-II ab48394 1 : 1000; Anti-GAPDH ab181602 1 : 10000) at 4°C overnight. Membranes were incubated with horseradish peroxidase-conjugated to secondary antibody at room temperature. Bands were visualized using clarity enhanced chemiluminescence kit (Bio-Rad, CA, USA) and were analyzed using Bio-Rad image Lab software, are data presented in arbitrary units (AU).

### 2.4. Histology

Fresh kidney from each mouse was fixed in 4% formaldehyde and embedded in paraffin. Then, 4-*μ*m thick sections were transferred to positive charged glass slides and processed for Hematoxylin and Eosin (H&E) staining. Images were captured using an Axio Lab. A1 microscope. Images were captured with Axiocam 105 color (ZEISS) and analyzed with ZEISS ZEN software. Photograph analysis presents data at pixels (glomerular size) or % of stained tissue (the rest of the histological parameters).

### 2.5. Immunohistochemistry

Renal tissue histological slides underwent immunohistochemical staining by incubating with primary antibodies against ATG5 (Anti-APG5L/ATG5 ab109490 1 : 100), LC3B (LC3B ab48394 1 : 200), and fibronectin (Fibronectin ab2413 1 : 100) in blocking solution (CAS-block, 8120, Invitrogen, UK).

HRP-polymer antirabbit (Nichirei and Dako) was used as a secondary antibody, and proteins were visualized using the DAB Plus Substrate System (TA-125-HDX, Thermo Scientific, USA) and then counterstained with Hematoxylin. Images were taken at 20× and 10× by using an Axio Lab. By using an Axio Lab, a 1 microscope with the Axiocan 105 color digital camera and ZEIN software (Zeis, Oberkochen, Germany), photograph analysis presents data as % of stained tissue.

### 2.6. Statistics

All results are reported as mean ± SEM. After conforming data normality distribution and equal variation among groups, comparisons between the study groups were performed using ANOVA, in GraphPad prism version 5.00 for windows (GraphPad Software, La Jolla, CA, USA). *P* value < 0.05 was considered statistically significant.

## 3. Results

### 3.1. Mice General Parameters

**Figure 1:**
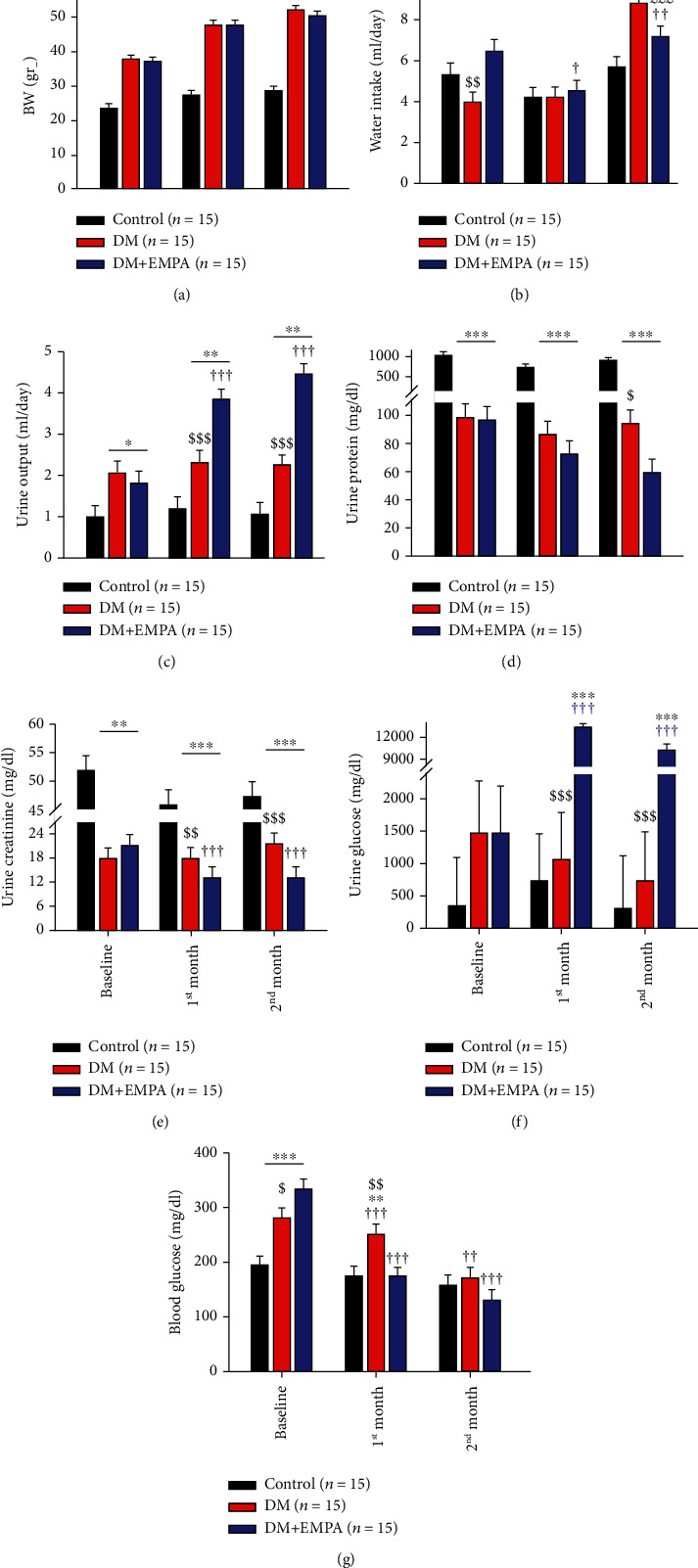
Mice general maintenance physiological parameters. (a) Mean weight, (b) water intake, (c) urine output, (d) urine protein exertion, (e) urine creatinine, and (f) urine glucose level. C57Bl/6 group (black columns), DM mice (red columns), and DM+EMPA (blue columns). ^∗^*P* < 0.05 vs. C57Bl/6, ^∗∗^*P* < 0.01 vs. C57Bl/6, ^∗∗∗^*P* < 0.001 vs. C57Bl, $*P* < 0.05 vs. DM+EMPA, $$*P* < 0.01 vs. DM+EMPA, $$$*P* < 0.001 vs. DM+EMPA, £££*P* < 0.001 vs. one month, †*P* < 0.05 vs. BL, ††*P* < 0.01 vs. BL, †††*P* < 0.001 vs. BL. (g) Mice blood glucose level. C57 group (black columns), DM mice (red columns), and DM+EMPA (blue columns). ^∗∗^*P* < 0.01 vs. C57, ^∗∗∗^*P* < 0.001 vs. C57, $*P* < 0.05 vs. DM+EMPA, $$*P* < 0.01 vs. DM+EMPA, ††*P* < 0.01 vs. BL, †††*P* < 0.001 vs. BL. Data represent the mean ± SEM.

At baseline (BL), the average DM and DM+EMPA mice BWs were 37.6 ± 0.9 gr., and the C57/Bl mice BW was 23.4 ± 0.9 gr., and consistently throughout the study, the C57/Bl mice were leaner than the DM and the DM+EMPA mice (Figure 1(a)). The mice consumed 3.9 ± 0.5 ml/day, 6.4 ± 0.5 ml/day, and 5.3 ± 0.5 ml/day water in the DM, DM+EMPA, and C57/Bl groups, respectively; though EMPA was not yet administered, higher water consumption was found in the DM+EMPA group (Figure 1(b)). Initial urine outputs for the DM and the DM+EMPA groups were 2.07 ± 0.26 ml/day and 1.81 ± 0.26 ml/day, respectively. The C57BL/6 group urine output was significantly lower, 0.98 ± 0.26 ml/day, (*P* < 0.05 vs. both DM groups, Figure 1(c)).

At one and two months, the DM mice (DM and DM+EMPA groups) gain weight considerably (*P* < 0.01 vs. BL and C57BL/6 (Figure 1(a)). Water intake was preserved at one month in all groups, but increased significantly at 2 months in the DM group and DSM+EMPA group vs C57BL/6 (*P* < 0.001, Figure 1(b)). Urine output of the DM mice was preserved at 2.32 ± 0.26 ml/day and 2.26 ± 0.26 ml/day at one and two months, respectively (*P* > 0.05, Figure 1(c)). Yet, for the DM+EMPA mice group, urine output increased as soon as one month of treatment reaching 3.84 ± 0.26 ml/day and 4.45 ± 0.26 ml/day at one and two months, respectively (*P* < 0.001, Figure 1(c)). Urine composition, i.e., urine protein, creatinine, and glucose, was all preserved along the experiment in the DM and C57BL/6 groups; yet, C57BL/6 mice consistently had higher values of protein and creatinine in their urine (*P* < 0.01, Figures 1(d) and 1(e)).

DM+EMPA group's urine composition changed dramatically at one and two months of experiment (Figures 1(d)–1(f)). Specifically, the DM+EMPA basal urine protein level of 95.9 ± 64.4 mg/dl was reduced to 72.7 ± 9.7 mg/dl at one month and to 58.9 ± 9.7 mg/dl at two months (*P* < 0.001 vs. C57BL/6 (Figure 1(d)). This group's basal urinary creatinine (21.4 ± 2.8 mg/dl) was decreased to 12.8 ± 2.8 mg/dl at one month and preserved at 13.2 ± 2.8 mg/dl at two months (*P* < 0.001 vs. C57/Bl and *P* < 0.01 vs. DM) (Figure 1(e)). BL DM+EMPA urine glucose (1484.6 ± 727.6 mg/dl) which was comparable at BL to the other groups increased to 13038.5 ± 702.9 mg/dl and 10402 ± 702.9 mg/dl at one and two months, respectively (*P* < 0.001 vs. C57BL/6 and DM, Figure 1(f)).

Blood glucose level at BL was 193.7 ± 16.2 mg/dl, 280.0 ± 16.2 mg/dl, and 333.9 ± 16.2 mg/dl for the C57B/6, DM, and DM+EMPA groups, respectively (Figure 1(g), *P* < 0.001 C57BL/6 vs. DM and DM+EMPA, and *P* < 0.05 DM vs. DM+EMPA). C57/Bl group preserved its blood glucose level at one and two months, 173.9 ± 16.8 md/dl and 157.9 ± 16.2 mg/dl, respectively (Figure 1(g)). In the DM group, blood glucose decreased with time to 250.9 ± 16.8 mg/dl and 170.5 ± 16.2 mg/dl, at one and two months, respectively. In the DM+EMPA group, blood glucose decreased dramatically to 173.7 ± 16.8 mg/dl and 129.7 ± 16.2 mg/dl, respectively (Figure 1(g)).

### 3.2. Differences in Renal Morphology

**Figure 2:**
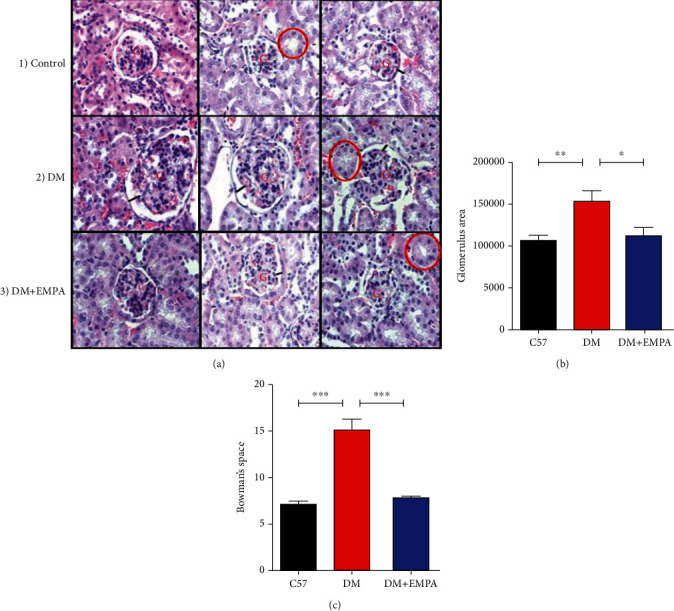
(a) Morphological H&E staining of kidney sections. A1. Representative H&E staining of healthy mice presents intact kidney morphology manifested with normal glomerulus size (red G), proximal convolute tubule (red circle), and Bowman's capsular space (black line). A2. Enlargement of glomerulus size and relatively wider Bowman's capsular space shown in representative morphology staining of DM mice. A3. Representative staining of DM+ EMPA presents reduced glomerulus size and relatively narrow Bowman's capsular space and thicker Bowman membrane. (b) Total analysis quantitative measurement of glomerular size in the three groups and (c) Bowman's capsule space size summary in the three groups.

Kidney sections of C57BL/6 mice (Figure 2(a)) present normal glomerulus size and Bowman capsular space. Enlarged glomerulus and a relatively wider Bowman capsular space were shown in kidney sections of DM mice (Figure 2(a)). Glomerulus size and Bowman capsular space width were reduced in kidney section of DM+EMPA mice which resembled C57BL/6 mice (Figure 2(a)). Indeed, quantification of glomerular size in DM mice (153813 ± 38977 pixels) was increased compared with C57BL/6 mice (105303 ± 23885 pixels, *P* < 0.01) and decreased in the DM+EMPA group (110505 ± 399326 pixels, *P* < 0.05, Figure 2(b)). Bowman's space area increase significantly in DM mice (15.0 ± 4.3%) compared to C57BL/6 (7.1 ± 1.6%, *P* < 0.001, Figure 2(b)) and was preserved in EMPA treatment compared with untreated DM mice (7.1 ± 1.6%, *P* < 0.001, Figure 2(c)).

**Figure 3:**
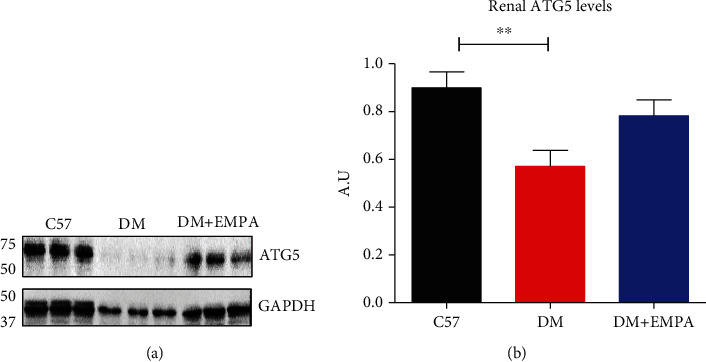
Renal ATG5 expression level in healthy control and DM and DM+EMPA mice. (a) Representative randomly Western blots of ATG5 protein levels in renal lysates. (b) The signals were quantified by densitometry, and the ratio between ATG5 and GAPDH signals in the three groups was calculated.

**Figure 4:**
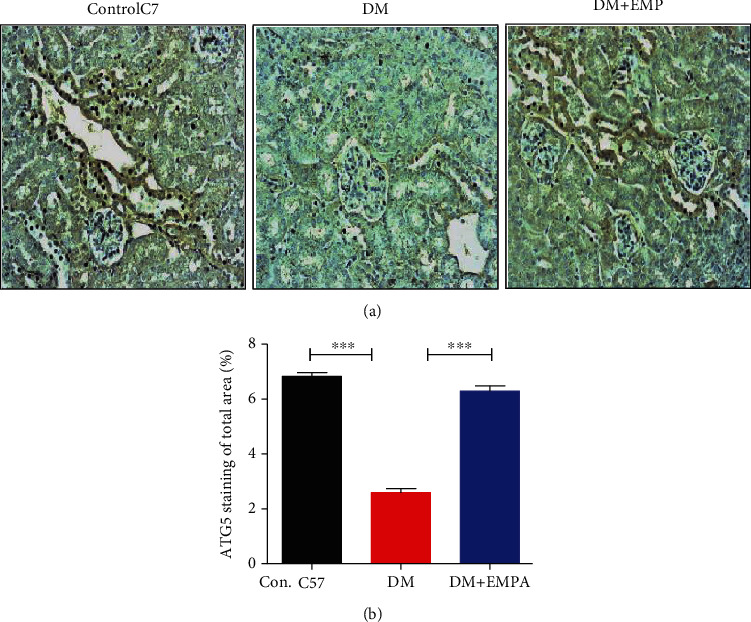
(a) Representative IHC staining of ATG5 in mice renal sections (magnification ×20). (b) Quantification of total renal ATG5 expression in IHC staining: renal ATG5 was significantly decreased in DM mice compared with C57/Bl mice. A significant increase in ATG5 levels was observed in DM+EMPA mice compared with DM mice.

ATG5 protein levels decreased significantly in DM mice compared with C57/Bl mice, and ATG5 expression was intermittent in DM+EMPA mice (Figure 3(a)). Quantification of the Western blots indicates significantly reduced ATG5 level in renal lysate of DM mice compared with C57BL/6 mice, 0.57 ± 0.25 AU and 0.90 ± 0.25 AU, respectively (*P* < 0.01, Figure 3(b)), and higher yet nonsignificant level was found in the DM+EMPA treatment, 0.78 ± 0.26 AU (Figure 3(b)). In histological analysis, ATG5 expression in renal tubules of DM mice was lower than of C57BL/6 and DM+EMPA (Figure 4(a)). Photograph analysis confirm that renal ATG5 was significantly decreased in DM mice compared with C57BL/6 or DM+EMPA, 2.6 ± 0.5%, 6.8 ± 0.5%, and 6.3 ± 0.8%, respectively (*P* < 0.001, Figure 4(b)).

**Figure 5:**
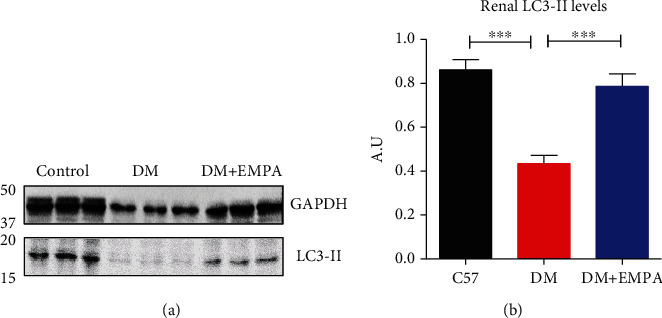
Renal LC3-II protein levels in C57/Bl, DM, and DM with EMPA lysates. (a) Representative randomly selected Western blots of LC3-II protein. (b) The signals were quantified by densitometry, and the ratio between LC3-II to GAPDH signals in the three groups was calculated.

LC3-II protein levels decreased in DM mice compared with C57/Bl. Higher protein level was observed in kidney lysate of the DM+EMPA-treated mice (Figure 5(a)). The measurement of renal LC3-II level confirms that its level was significantly reduced in DM mice compared with C57BL/6 or DM+EMPA, 0.44 ± 0.14 AU, 0.86 ± 0.18 AU, and 0.78 ± 0.22 AU (Figure 5(b), *P* < 0.001).

**Figure 6:**
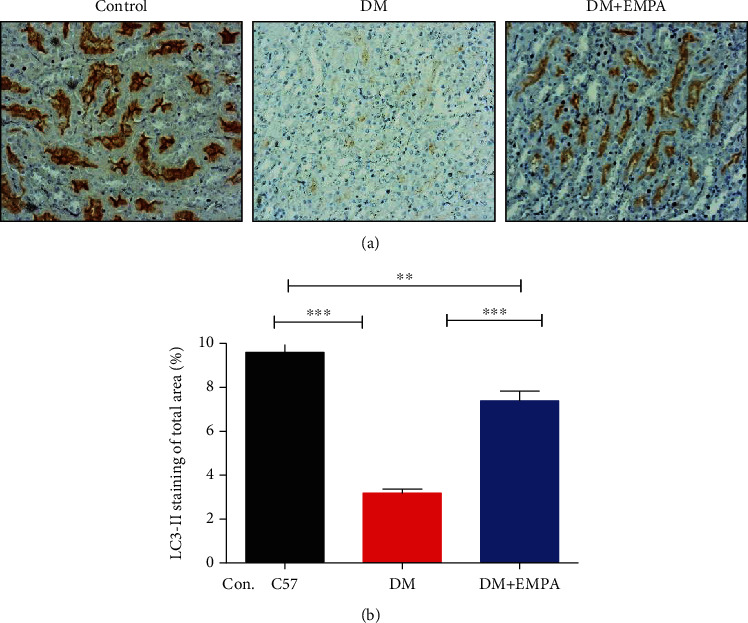
(a) Representative IHC staining of LC3-II in mice renal sections (magnification ×20). (b) Quantification of total LC3-II expression-renal LC3-II was significantly decreased in DM mice compared with c57/Bl mice. Higher than DM and similar to C57/Bl level, the expression of LC3-II was observed in DM+EMPA mice compared with DM mice.

Concomitantly, in immune-histochemical analysis LC3-II expression in renal tubules of untreated DM mice was dramatically reduced compared with C57BL/6 control, and DM+EMPA mice presented higher LC3-II expression than in the DM (Figure 6(a)). The quantification of renal LC3-II expression indicate significant reduction in DM mice compared with C57/Bl or DM+EMPA group, 3.2 ± 0.7%, 9.6 ± 2.1%, and 7.4 ± 1.7% (*P* < 0.001, Figure 6(b)). Nevertheless, the LC3-II expression in the DM+EMPA was still lower than the C57BL/6 (*P* < 0.01, Figure 6(b)).

**Figure 7:**
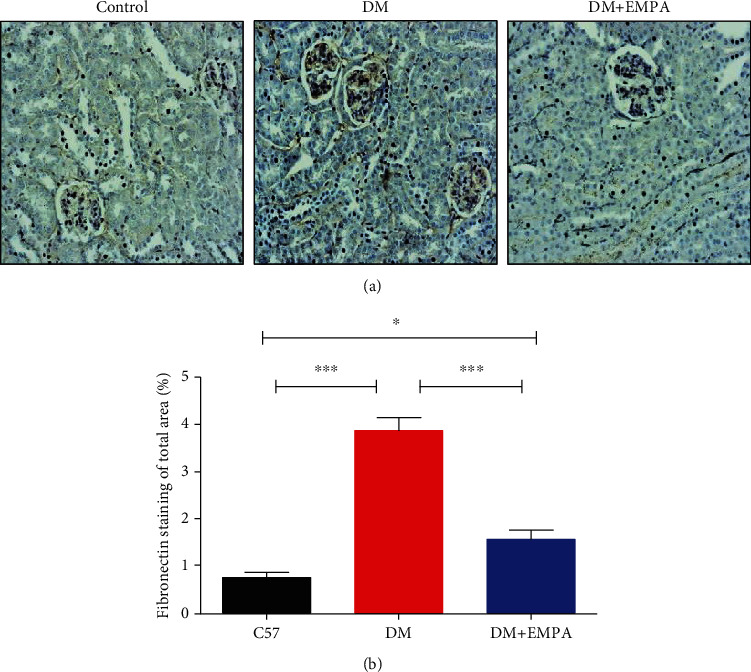
(a) Representative IHC staining for fibronectin in mice renal sections (magnification × 20). (b) Quantification of fibronectin expression. Renal fibronectin expression was significantly increased in DM mice compared with C57/Bl and DM+EMPA mice.

Fibronectin expression, as a measure of renal fibrosis, was evaluated in the glomeruli of the histological specimens. In Figure 7(a), fibronectin expression is presented, and specifically, high expression is seen in DM renal sections compared with CB57/Bl group. EMPA-treated mice presented decreased fibronectin expression compared with DM mice (Figure 7(a)). Quantification of fibronectin expression indicates increase of protein abundance in DM mice group vs. C57/Bl or DM+EMPA group, 3.9 ± 1.1%, 0.8 ± 3.8%, and 1.6 ± 0.8%, respectively (*P* < 0.001, Figure 7(b)). Interestingly, at the DM+EMPA group, still fibronectin expression was higher than in the C57BL/6 group (*P* < 0.05, Figure 7(b)).

## 4. Discussion

DN presents a significant morbidity and mortality burden, and until recently, treatment options have been limited to risk factor control such as hypertension and hyperglycemia by RAAS inhibition and standard antidiabetic drugs [14–17]. Recently, different studies have concentrated in other pathways involved in DN prevention and progression such as autophagy, and other modalities of treatments that confer not only glycemic effect but also reno-protective effects were tested [14–18].

Autophagy pathway is crucial for cell survival, differentiation, development, and homeostasis under stress conditions such as hyperglycemia [19, 20]. Various studies have provided evidences suggesting that autophagy-related key proteins ATG5 and LC3-II play a critical role in a variety of disease processes such as DM [19–22]. Yet only few have examined the possible role of ATG5/LC3-II proteins in the development and progression of DM and its vascular complication such DN [22, 23, 38–40]. We analyzed the ATG5 protein that dissociates from the membrane next to the completion of autophagosome formation, and the LC3-II protein, one of the most frequently used biomarkers for autophagy. LC3-II is a gold standard marker for tracing changes in the autophagy process [24, 38–41]. ATG5 might play a crucial role in the production and secretion of insulin in pancreatic *β* cells, and deletion of ATG5 results impaired insulin secretion and glucose intolerance [42]. In recent years, autophagy has become a hot topic in the field of DN, suggesting that it is likely to be a key target for the prevention and treatment of DN. Yet, the activity of autophagy in the renal cells and changes in the autophagy-lysosomal system during DN is still under debate [41, 43–45]. Hyperglycemia was considered an autophagy suppressor in the glomerular, proximal convolute tubules, podocytes and mesangial cells [43].

The emergence of SGLT2i such as EMPA, a new class of glucose-lowering compounds, has shown not only blood glucose lowering effect, but also pleiotropic effects with reno-protective actions in preclinical and human studies [27]. In the present study, we provide novel mechanistic evidence showing that EMPA improves the autophagy process activities in the glomerular cells and renal proximal tubular cells, all of which led to reduced renal fibrosis caused by hyperglycemia. The acceleration of autophagy flux under EMPA treatment is associated with regression of glomerular sclerosis and preservation of glomerular (podocytes) morphology, as well as with a slowdown in the growth of albuminuria.

Various studies have assessed the impact of DM on the reno-protective autophagy processes on renal cells exposed to hyperglycemia [22, 45]. Anton I. Korbut et al. demonstrate that EMPA as a monotherapy and in combination with other antidiabetic drugs; enhanced the areas of glomerular staining for beclin-1, LAMP-1, LC3-II, and BcL-2; and increased volume density of autophagosomes and autolysosomes in podocytes. All together indicate restoration of autophagy by EMPA. Further, the data provide explanation for the mechanism of the reno-protective effect of SGLT2i and possibly DPP4 inhibitors as well in diabetes.

Here, we report that DM mice exhibit significantly decreased levels of ATG5 and LC3-II protein levels compared to healthy control mice, which restored to near normal with EMPA treatment. We used immunohistochemistry on mouse tissue sections as well as Western blot analysis. Our data suggest that there is a link between ATG5-LC3-II dysregulation and DM. These results correlate with previous studies, which showed that alterations in ATG5/LC3 expression are implicated in many pathological conditions, including DM [26, 43]. Restoration of the autophagy process, decreased glomerular hypertrophy, and regression of fibrosis in diabetic mice in this model identifies an unappreciated potential benefit of SGLT2i in patients with DN.

## 5. Conclusions

DN patients have increased risk of morbidity and mortality. Hyperglycemia is a modifiable risk factor for cardiovascular complications and progression of DN. There is now conclusive evidence and consensus that SGLT2i significantly reduce progression of DN and onset of end-stage renal disease, stroke, heart attack, and death in T2DM [46].

In our study, EMPA treatment decreases blood glucose levels in the T2DM mice with increased excretion of glucose in the urine. Both ATG5 and LC3-II protein levels decreased in those mice compared to control mice and increased following EMPA treatment. Our findings may be translated into clinical practice approach and may lead to further studies to address DM and its vascular complications by selective modulation of ATG5/LC3 expression with new agents such as SGLT2i and newer medications such as DPP4 inhibitors [46–48].

## Data Availability

The data analyzed during the study are not publicly available. Rigorous analysis of the data has been performed in order to ensure the objective authenticity of the results.

## Abbreviations

DM: Diabetes mellitus
DN: Diabetic nephropathy
DR: Diabetes retinopathy
GFR: Glomerular filtration rate
ER: Endoplasmic reticulum.
